# An Age-stratified, Randomized Immunogenicity Trial of Killed Oral Cholera Vaccine with Delayed Second Dose in Cameroon

**DOI:** 10.4269/ajtmh.22-0462

**Published:** 2022-11-14

**Authors:** Jérôme Ateudjieu, David A Sack, Sonia Sonkeng Nafack, Shaoming Xiao, Ketina Hirma Tchio-Nighie, Herve Tchokomeni, Landry Beyala Bita’a, Paul Ntsekendio Nyibio, Etienne Guenou, Kedia Mayah Mondung, Frank Forex Kiadjieu Dieumo, Rosanne Minone Ngome, Kelsey N. Murt, Malathi Ram, Mohammad Ali, Amanda K. Debes

**Affiliations:** ^1^ MA Sante, Yaoundé, Cameroon;; ^2^ Department of Public Health, Faculty of Medicine and Pharmaceutical Sciences, University of Dschang, Cameroon;; ^3^ Clinical Research Unit, Division of Health Operations Research, Ministry of Public Health, Cameroon;; ^4^ Department of International Health, Johns Hopkins Bloomberg School of Public Health, Baltimore, Maryland;; ^5^ Department of Bacteriology-Parasitology-Mycology Laboratory, Centre Pasteur of Cameroon (CPC), Yaoundé, Cameroon

## Abstract

The recommended schedule for killed oral cholera vaccine (OCV) is two doses, 2 weeks apart. However, during vaccine campaigns, the second round is often delayed by several months. Because more information is needed to document antibody responses when the second dose is delayed, we conducted an open-label, phase 2, noninferiority clinical trial of OCV. One hundred eighty-six participants were randomized into three dose-interval groups (DIGs) to receive the second dose 2 weeks, 6 months, or 11.5 months after the first dose. The DIGs were stratified into three age strata: 1 to 4, 5 to 14, and > 14 years. Inaba and Ogawa vibriocidal titers were assessed before and after vaccination. The primary analysis was geometric mean titer (GMT) 2 weeks after the second dose. Data for primary analysis was available from 147 participants (54, 44, and 49 participants from the three DIGs respectively). Relative to the 2-week interval, groups receiving a delayed second dose had significantly higher GMTs after the second dose. Two weeks after the second dose, Inaba GMTs were 55.1 190.3, and 289.8 and Ogawa GMTs were 70.4, 134.5, and 302.4 for the three DIGs respectively. The elevated titers were brief, returning to lower levels within 3 months. We conclude that when the second dose of killed oral cholera vaccine was given after 6 or 11.5 months, vibriocidal titers were higher than when given after the standard period of 2 weeks. This provides reassurance that a delayed second dose does not compromise, but rather enhances, the serological response to the vaccine.

## INTRODUCTION

Cholera is an infectious disease that continues to cause outbreaks with a high burden in countries of Asia, Africa, and, until recently, in Hispaniola.^1^ It occurs most often in areas without adequate water and sanitation when the pathogen, *Vibrio cholerae*, is introduced into the area. The intestinal infection with *V. cholerae* causes diarrhea, which in severe cases, leads to severe, life-threatening dehydration. Deaths can be prevented with rehydration using oral or intravenous rehydration, but outbreaks may occur suddenly, before facilities are prepared to provide adequate treatment or when patients are not able to reach the health facility. This contributes to higher case fatality ratios early in outbreaks or in areas without access to treatment.^2^^,^^3^ Furthermore, health systems of most vulnerable areas are characterized by limited access to water, sanitation, and hygiene (WASH), insufficient preparedness of health facilities and weakness of the epidemiological surveillance system.^4^ Cholera vaccination, using the killed oral cholera vaccine (OCV) can reduce risk of cholera in areas that are prone to outbreaks.^5^^–^^7^ Two WHO prequalified brands of the killed OCV are available through the global stockpile: Shanchol (Shantha Biotechnics Limited, Telangana, India) and Euvichol (Eubiologics, Chunceon, South Korea). When used during vaccine campaigns, two doses of the vaccine are recommended for all persons ≥ 1 year of age, with the doses to be given 2 weeks apart.^8^

Although OCV is effective against cholera, it must be used strategically and be given to people at highest risk. This includes 1) preventive use for people living in geographic areas deemed to be cholera hotspots, 2) preventive and emergency use for people in a humanitarian crisis in areas with risk of cholera, and 3) emergency use to control an outbreak.^8^ Unfortunately, the supply of OCV is limited, and this restricts the number of doses that can be distributed, emphasizing the need for strategic use.^7^^,^^9^ There is evidence that, under some circumstances, a single dose is also effective at least for the short term^10^^,^^11^ and that use of a single dose might be appropriate^12^ or, alternatively, that a single dose may be used to vaccinate a larger number of people with a single dose and then a second dose could be administered later when additional vaccine doses are available or when local resources allow for this.^13^^,^^14^

If there is to be a delay in administering the second round of vaccine, the acceptable interval for this delay is not known. Changing the interval to 1 month was not inferior to 2 weeks,^15^ but in some cases, the second round of the campaign has been longer than 6 months.^13^ There is also evidence that giving a booster dose after a prolonged period can lead to an augmented response,^16^ suggesting there could be a benefit in delaying the second dose. A study in Zambia found that a second dose given at 6 months is not inferior,^17^ but the results from the Zambia study were not known when planning the Cameroon study. It was also felt that additional data, including a group receiving the vaccine after an even longer interval, was needed to guide policy decisions regarding acceptable dose intervals.

The present study was conducted to compare the vibriocidal antibody titers when the two doses of OCV were given at longer intervals; thus, participants were randomized to receive the second dose of OCV after 2 weeks, 6 months, or 11.5 months after the first dose. We then determined the geometric mean titers (GMT) 2 weeks after the second dose, as well as additional follow-up periods in the different groups. We hypothesized that the serum vibriocidal GMTs in serum obtained 2 weeks after the second dose would not be inferior to the recommended interval of 2 weeks if the second dose was extended to 6 months or 11.5 months. Additional aims of the study included an evaluation of 1) age-group-specific serum vibriocidal GMTs during the same time intervals, 2) vibriocidal seroconversion rates in the participants overall and by age group, and 3) persistence of elevated vibriocidal GMTs during follow-up for several months. Because protection from OCV is age dependent,^18^ we stratified participants into three age strata (1–4 years, 5–14 years, and > 14 years). We understand that this study is not able to compare effectiveness of protection against cholera with these different dose intervals; nevertheless, the vibriocidal titer after vaccination is associated with protection against cholera.^19^^,^^20^

## METHODS

### Ethical considerations.

This study was approved by the Cameroon National Research Ethics Committee (2018/02/975/CE/CNERSH/CE) and by the Institutional Review board of the Johns Hopkins Bloomberg School of Public Health (IRB No. 8114). Potential study participants received full information regarding study objectives, procedures, duration, and their rights to decide to participate or not to participate. Adults gave signed informed consent and parents of young children (1–12 years) provided signed parental permission. Parental permission and participant assent was obtained for participants aged 13 to 20 years, The trial was monitored by a local monitor, and the protocol was registered at Clinical Trials.gov (NCT03719066).

### Study design and participants.

This was an open label, randomized, phase 2, noninferiority clinical trial. Participants were randomly allocated by stratified random sampling to three dose-interval groups (DIGs). Each DIG received two doses of OCV with the first dose given on day 0. The second dose was given 2 weeks later for DIG1, at 6 months for DIG2, and at 11.5 months DIG3. Each DIG was divided into three age strata: 1 to 4 years, 5 to 14, and > 14 years. Vibriocidal titers were assessed at different time points before and after vaccination. Acceptable time windows were defined for the dose intervals and blood draws.

The study was conducted at the Soboum health area situated in the Nylon health district in the large port city of Douala, Cameroon. This is a crowded urban area with working-class families. Most people living in this area are involved in commercial and urban transport activities, and a smaller fraction working in business and as civil servants.

The first dose was administered on October 26, 2018, and the field phase of the study ended on February 27, 2020. Households of the health area were visited by trained community volunteers to invite potential participants to the study site. Enrollment was done at the Soboum Medical Center, which is the main health facility for delivering primary health care in the Soboum health area. Although this area is considered at risk for cholera, there were no cases reported in Douala since a large outbreak in 2010–2012. OCV had never been used in the study area before or during the study period other than through this study.

Residents of the Soboum health area aged ≥ 1 year were eligible to participate. We included clinically healthy participants who assured the study team that they would be available during the study period and consented to participate. At the time of recruitment and before the second dose, we excluded pregnant women, those who received OCV from other sources or who received an investigational product (within 30 days before vaccination). We also excluded those with a history of diarrhea during the 7 days before first dose of vaccine (defined as ≥ 3 unformed loose stools in 24 hours); history of chronic diarrhea (lasting for more than 2 weeks in the past 6 months); current use of laxatives, antacids, or other agents to lower stomach acidity; planning to become pregnant in the next 2 years; presence of a significant medical or psychiatric condition. Examples include reported diagnosis and treatment of tuberculosis or HIV, renal insufficiency, hepatic disease, oral or parenteral medication known to affect the immune function such as corticosteroids or other immunosuppressant drugs, or behavioral or memory issues. Participants who at one point could not been reached or did not present to the health facility for their second dose with no definitive reason were considered lost to follow-up and excluded from the analysis.

### Interventions.

**Figure 1.**
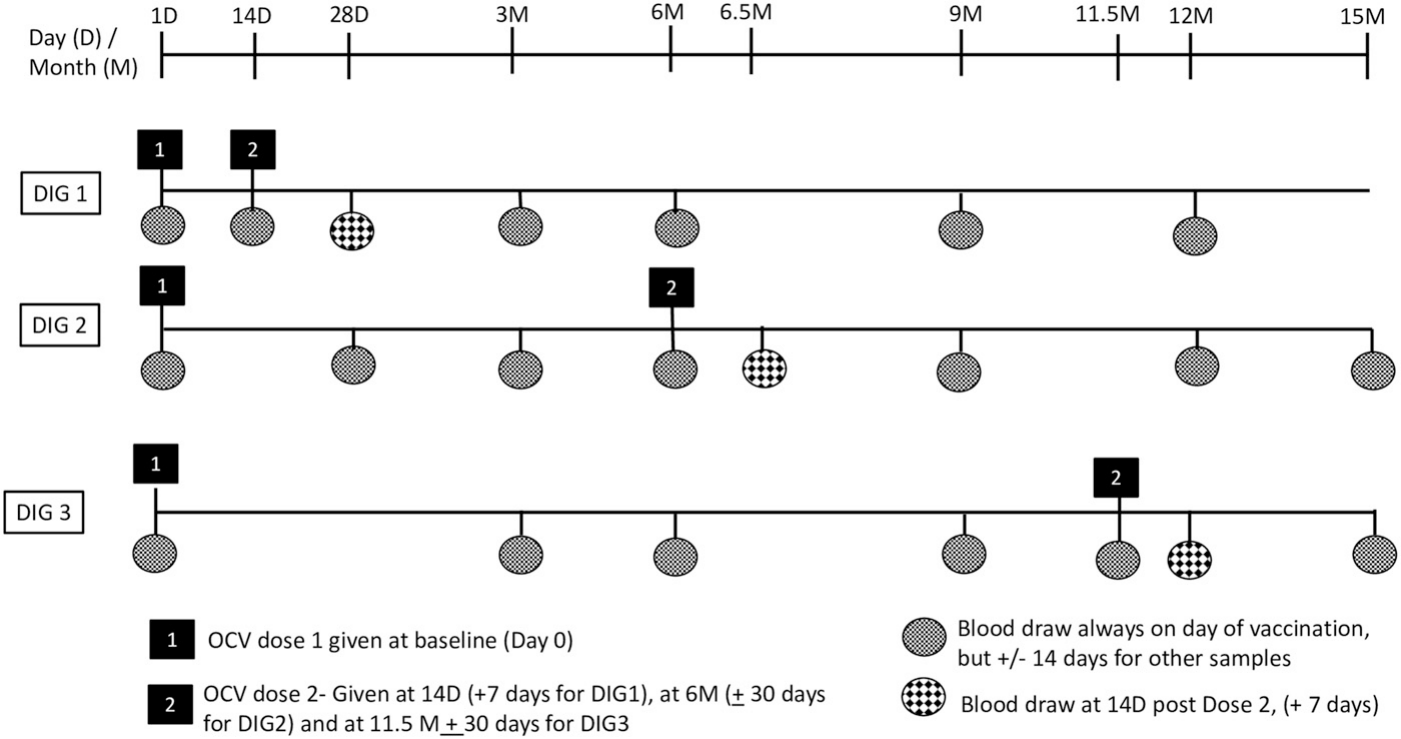
Event schedule for the dose interval groups receiving killed oral cholera vaccine in Cameroon. DIG = dose-interval group; OCV = oral cholera vaccine.

The cholera vaccine used in the study was Shanchol (Lot 2SCNO33A17), which consists of a mixture of killed *V. cholerae* O1 and O139 in single-dose glass vial containing 1.5 mL of vaccine. The entire vial of OCV was administered orally to each participant irrespective of their ages. The vaccine vial was shaken to ensure mixing and was inspected by the study nurse before administration. To monitor for safety, all participants were observed for 30 minutes after taking the vaccine. The timing of the follow-up vaccinations and blood draws along with the acceptable visit windows for each event are shown on Figure 1.

### Outcomes.

The participants were followed for 12 months for DIG1 and for 15 months for the other two DIGs. The primary outcome was the vibriocidal GMT measured 2 weeks after the second doses of OCV for each DIG. Secondary outcomes included 1) vibriocidal antibody seroconversion rates (≥ 4-fold increase in vibriocidal titer compared with the baseline titer) 2 weeks after the second dose, 2) age-group-specific serum vibriocidal GMTs and seroconversion rates 2 weeks after the second dose in each DIG. Additional outcomes compared the GMTs at other time points to determine persistence of the vibriocidal titer changes and the geometric mean titer fold increase ratio.

### Laboratory procedures.

For adults, 5 mL of whole blood was collected by venipuncture. For children (1–4 years), 350 µL capillary blood was collected using a capillary tube and diluted 1:5 in normal saline. The blood samples were transported with ice packs to the field laboratory at the Congo medical center located at about 5 km away from the Soboum Medical Center. The samples were centrifuged for serum extraction and stored at –20°C and were then transported to the central laboratory based in Yaoundé, ∼240 km from Douala for serologic analyses.

The serum vibriocidal responses were measured as previously described,^17^^,^^21^ except that *V. cholerae* strains used as the target vibrio strains were originally isolated in Cameroon (Inaba FO14-2018-EN and Ogawa CPC-BACT-01). Briefly, the strains were incubated with serially diluted, heat-inactivated serum and exogenous guinea pig complement (Sigma Aldrich, catalogue # S234395) on a shaker (50 rev/min) at 37°C for 1 hour.^16^ The starting dilution for the previously undiluted serum was 1:10 while the previously diluted serum from young children was used without further dilution considering the 1:5 dilution of whole blood to be equivalent to 1:10 dilution of serum. Vibriocidal titers were defined as the reciprocal of the highest serum dilution resulting in a 50% reduction in optical density (595 nm) compared with growth control without serum. Standard sera, both high titer human serum and high titer rabbit serum were used to normalize the results and control for inter-experimental variation. Samples were tested in duplicates. Titers lower than the lowest detectable titer were assigned to have a titer equivalent to half of the lowest detectable titer. The threshold for inter- and intra-experimental variation was set at 2-fold. Seroconversion was defined as ≥ 4-fold rise in vibriocidal titers compared with baseline titer.

### Statistical methods and randomization.

The sample size was calculated to determine noninferiority of the vibriocidal GMTs of DIG2 and DIG3 compared with DIG1 2 weeks after the second dose. The margin of noninferiority was set to 15%. The true difference between the GMTs was assumed to be 0.000. The data assumed a population with a standard deviation of 7.770 for both groups. With alpha = 0.025, beta 0.10, one-sided, two-sample mean, a sample size of 11 in each group was required. Assuming a 10% high baseline titer and 20% dropout over the study period, a sample size of 20 per age strata was calculated, totaling 180 subjects in the study.

Participants in each age strata were randomized into three DIGs using a block randomization of 6 per block. Before study initiation, three randomization tables for each of the age strata were generated using “Sealed Envelope” (https://www.sealedenvelope.com) to create a randomization list following the fixed block randomization method. The random assignment numbers were placed in sealed envelopes in sequential order and participants were given their DIG assignment based on their sequence of enrollment. Because the study was designed to include a given number of participants in each age stratum, recruitment continued to reach the planned number of participants, although in some cases a few additional participants were included for logistical reasons.

For the primary analysis, we included data from participants if they had taken both the first and second dose and had provided serum specimens for the primary outcome (baseline and two weeks after the second dose). Data was excluded from participants if they vomited one of the doses. Vomiting was defined as the expulsion of stomach contents through the mouth within 10 minutes after administration of the vaccine. None of the subjects in the study experiencing mild or transient spitting up when dosed. We observed each subject to ensure that each child swallowed the entire dose. If they missed one of the other scheduled blood draws, their available data were included when analyzing the “additional outcomes.”

All analyses were done between DIGs for overall and for each age group considering DIG1 as the reference group. Baseline characteristics and study outcomes were compared using χ^2^ test or Fisher exact test as appropriate for qualitative variables and Student’s *t* test for quantitative variables. Because of the imbalance of baseline titers, especially the higher baseline titers for DIG3, we calculated the geometric mean titer fold increase ratio by comparing the geomeans of the fold increase between baseline and follow-up sera. The 95% confidence interval of Geometric Mean Responses was constructed through 100,000 bootstraps. All analyses were conducted using R (version 4.0.4).

## RESULTS

### Enrollment.

**Figure 2.**
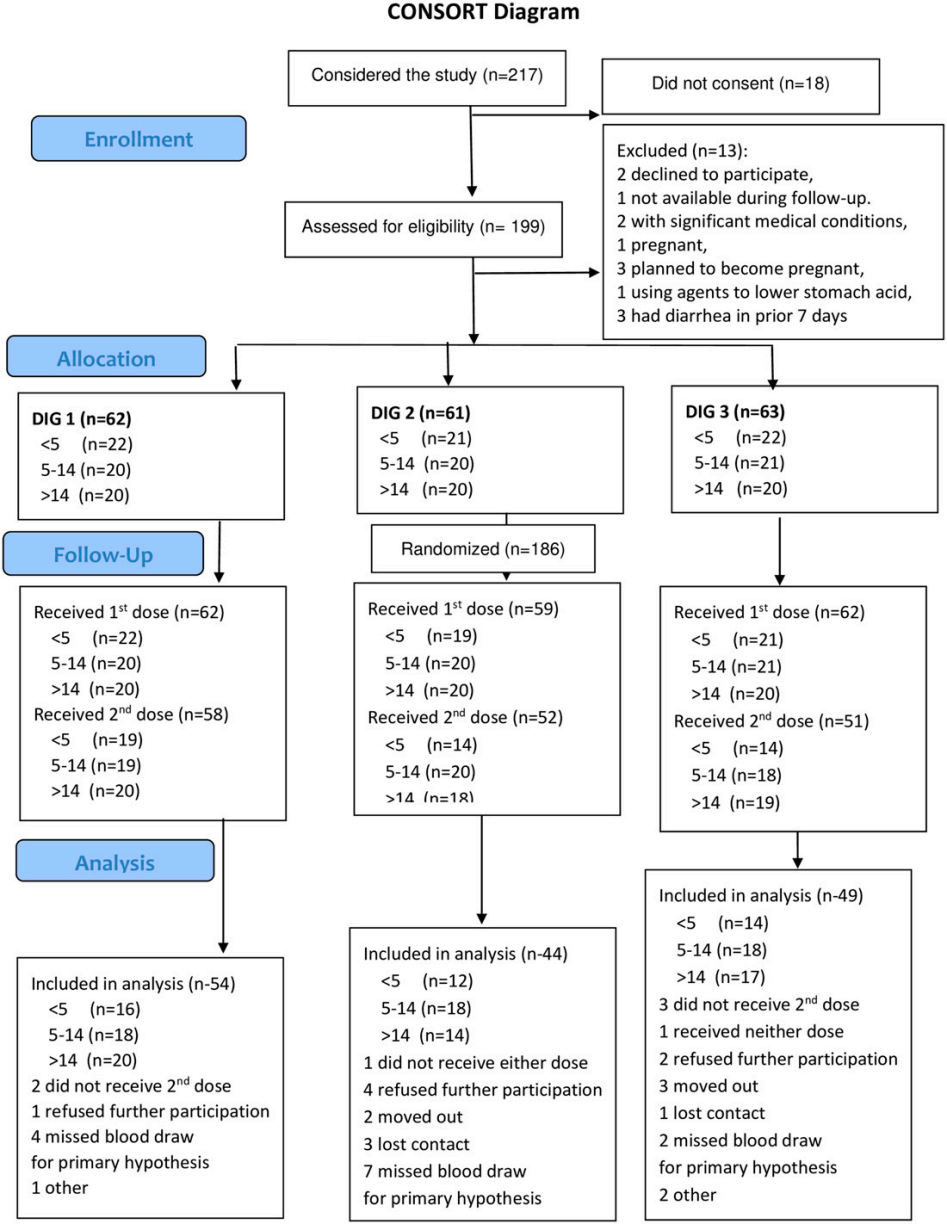
CONSORT flow diagram. DIG = dose-interval group. This figure appears in color at www.ajtmh.org.

Participants were engaged in the study from October 26, 2018, until February 27, 2020. Of 217 participants who came to the field study site to consider joining the study, 18 did not consent to be screened and 13 were excluded. One hundred and eighty-six (186) participants were enrolled, with 62, 61, and 63 randomly assigned to DIG1, DIG2, and DIG3, respectively. Two children in DIG2 and one child in DIG3 vomited a first dose of vaccine, and their data were excluded. As shown on the consort flow diagram, others did not take a second dose or missed a primary blood draw. Serum was available from 147 participants who took two full doses of vaccine and had blood samples 14 days after the second dose including 54, 44, and 49, respectively, in DIG1, DIG2, and DIG3. Data from these participants were included in the primary analysis. Details of participants flow are presented in the consort flow diagram of the study in Figure 2.

A comparison of participants’ characteristics per study DIG is presented in Table 1. The children in the study were well nourished with 38 of the 40 children in the 1 to 4 years age group having a weight-for-age z score within 1 z score of the reference value. The overall distribution of age groups, weight, and height did not differ significantly between DIGs, but there were significantly fewer males in DIG3. The groups did not differ significantly by type of housing, source of water, or type of toilet. The definition of improved toilet is consistent with the definition provided by the WHO.^22^

**Table 1:** Distribution and comparison of participant characteristics per study dose interval

	DIG1 (*N* = 54)	DIG2 (*N* = 44)	DIG3 (*N* = 49)	*P* value
Age group, *n* (%)				
1–4	16 (30.0%)	12 (27.3%)	14 (28.6%)	0.96
5–14	18 (33.0%)	18 (41.0%)	18 (36.7%)	
> 14	20 (37.0%)	14 (31.7%)	17 (34.7%)	
Sex, *n* (%)				
Male	23 (43.0%)	25 (56.8%)	15 (30.6%)	0.04
Weight (kg)	40.9 ± 25.2	40.8 ± 25.9	38.1 ± 25.2	0.82
Height (m)	1.35 ± 0.33	1.36 ± 0.32	1.30 ± 0.31	0.61
Main flooring material in house, *n* (%)				
Tiles	14 (26.0%)	16 (36.3%)	13 (26.5%)	0.74
Cement/brick	38 (70.3%)	26 (59.1%)	33 (67.4%)	
Mud/dirt	2 (3.7%)	2 (4.6%)	3 (6.1%)	
Main drinking water source, *n* (%)				
Own piped/borehole/tube well	18 (33.3%)	18 (40.9%)	10 (20.4%)	0.22
Piped/borehole/tube well from neighbor	32 (59.2%)	22 (50%)	33 (67.4%)	
Piped/borehole/tube well far from home	3 (5.6%)	4 (9.1%)	6 (12.2%)	
Unprotected well/pond/canal	0	0	0	
Other	1 (1.9%)	0	0	
Type of toilet facility, *n* (%)				
Improved	16 (29.7%)	12 (27.3%)	12 (24.5%)	0.84
Unimproved	38 (70.3%)	32 (72.7%)	37 (75.5%)	

DIG = dose-interval group.

### Baseline vibriocidal antibody titers.

As shown in Table 2, the baseline vibriocidal titers of the DIGs did not differ between DIGs except that the 5 to 14 years age group in DIG3 had more participants with an elevated Ogawa baseline titer (Table 3) and the baseline GMT for DIG3 was higher (Table 4).

**Table 2:** Baseline vibriocidal antibody titers by age group and arms

	1–4 years				5–14 years				> 14 years			
Baseline titers	DIG1, *N* = 16	DIG2, *N* = 12	DIG3, *N* = 14	*P* value	DIG1, *N* = 18	DIG2, *N* = 18	DIG3, *N* = 18	*P* value	DIG1, *N* = 20	DIG2, *N* = 14	DIG3, *N* = 17	*P* value
Inaba												
< 10	12	10	11	0.99	14	14	15	0.38	14	8	11	0.18
10–40	3	2	2		1	3	3		4	1	5	
≥ 40	1	0	1		3	1	0		2	5	1	
Ogawa												
< 10	15	11	12	0.91	13	17	9	0.03	14	9	6	0.14
10–40	1	1	1		3	0	6		2	4	6	
≥ 40	0	0	1		2	1	3		4	1	5	

DIG = dose-interval group.

**Table 3:** Vibriocidal GMTs and response rates in dose-interval groups before and 2 weeks after receiving oral cholera vaccine in Cameroon

	Inaba			Ogawa		
	DIG1 *N* = 54 (95% CI)	DIG2 *N* = 44 (95% CI)	DIG3 *N* = 49 (95% CI)	DIG1 *N* = 54 (95% CI)	DIG2 n-44 (95% CI)	DIG3 *N* = 49 (95% CI)
Baseline GMT	9.1 (6.1, 11.9)	9.7 (6.1, 13.0)	8.9 (5.8, 11.9)	8.1 (5.7, 10.4)	7.3 (4.8, 9.6)	17.1 (8.3, 25.0)
GMT 14 days after dose 2	55.1 (24.1, 82.5)	190.3 (76.1, 290.3)	289.8 (138.4, 423.8)	70.4 (35.6, 101.3)	134.5 (74.6, 188.5)	302.4 (159.5, 430.2)
GMF change from baseline	6.0 (3.1, 8.6)	19.6 (9.9, 28.4)	32.5 (15.1-47.8)	8.6 (4.2, 12.5)	18.4 (9.5, 26.4)	17.6 (7.6, 26.5)
GMFR		3.3 (1.1, 5.7)	5.4 (1.6, 9.2)		2.1 (0.5, 3.5)	2.4 (0.3, 3.7)
Vibriocidal response rate	24 (44.4%)	35 (79.5%)	40 (81.6%)	29 (53.7%)	34 (77.3%)	38 (77.6%)

CI = confidence interval; GMFR = geometric mean titer fold increase ratio; GMT = geometric mean titer.

**Table 4:** Age-specific seroconversion rates comparing DIG1 with groups receiving a delayed second dose

Age group	DIG1		RR	DIG2 and DIG3		RR
	Inaba					
	No. responding	Total		No. responding	Total	
1–4*	6	16	37.5%	20	26	76.9%
5–14	13	18	72.2%	30	36	83.3%
> 14**	5	20	25.0%	25	31	80.6%
Overall**	24	54	44.4%	75	93	80.6%
	Ogawa					
1–4*	6	16	37.5%	22	26	84.6%
5–14	12	18	66.7%	26	36	72.2%
> 14**	11	20	55.0%	24	31	77.4%
Overall**	29	54	53.7%	72	93	77.4%

DIG = dose-interval.

* *P* = 0.01.

** *P* ≤ 0.001, comparing response rates between DIG1 with DIG2 and DIG3.

### Comparison of GMTs between DIGs.

**Figure 3.**
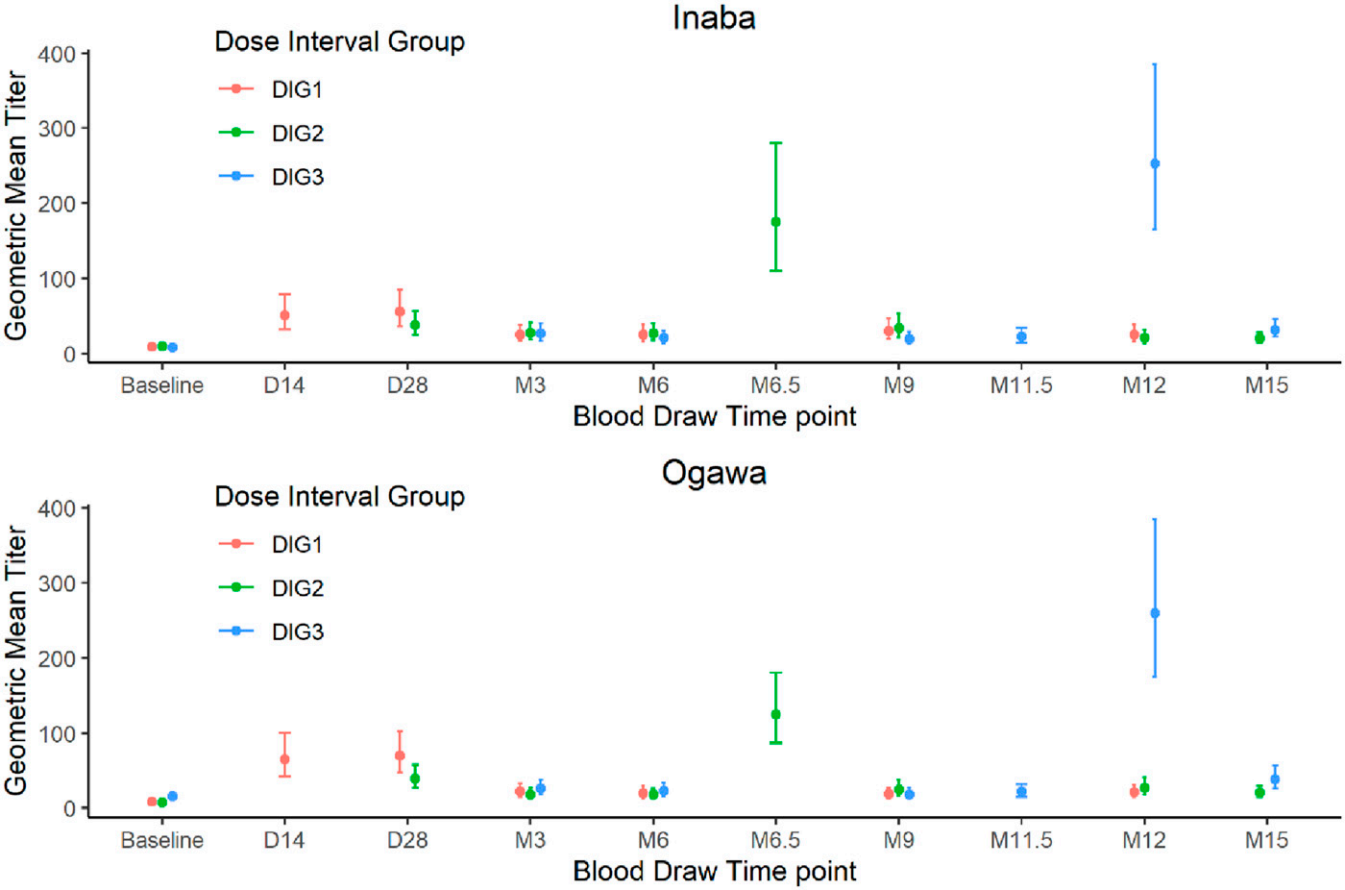
Vibriocidal geometric mean titers of the three dose interval groups through the span of the study. D = day; M = month.

The Inaba and Ogawa GMTs for the DIGs during the entire span of the study are shown in Figure 3. The titers were generally low except for the samples obtained 2 weeks after a dose. For DIG1, the GMT increased 2 weeks after the first dose and remained elevated 2 weeks after the second dose on day 28. As shown on Figure 3, giving a second dose 2 weeks after the first dose did not result in a further increase in GMT compared with GMT after a single dose. On day 28, the GMTs for DIG1 and DIG2 were not significantly different even though DIG1 had received two doses and DIG2 had received only a single dose by that time.

As expected, the vibriocidal GMTs 2 weeks after the second dose were all significantly higher than baseline GMTs; however, the increase in GMTs was greater for both DIG2 and DIG3 compared with DIG1. The vibriocidal GMTs before the first dose and 2 weeks after the second dose and response rates after the second dose are shown in Table 3. The follow-up titers 3 months after the second dose, although much lower than the peak, were significantly higher than the baseline GMT.

**Figure 4.**
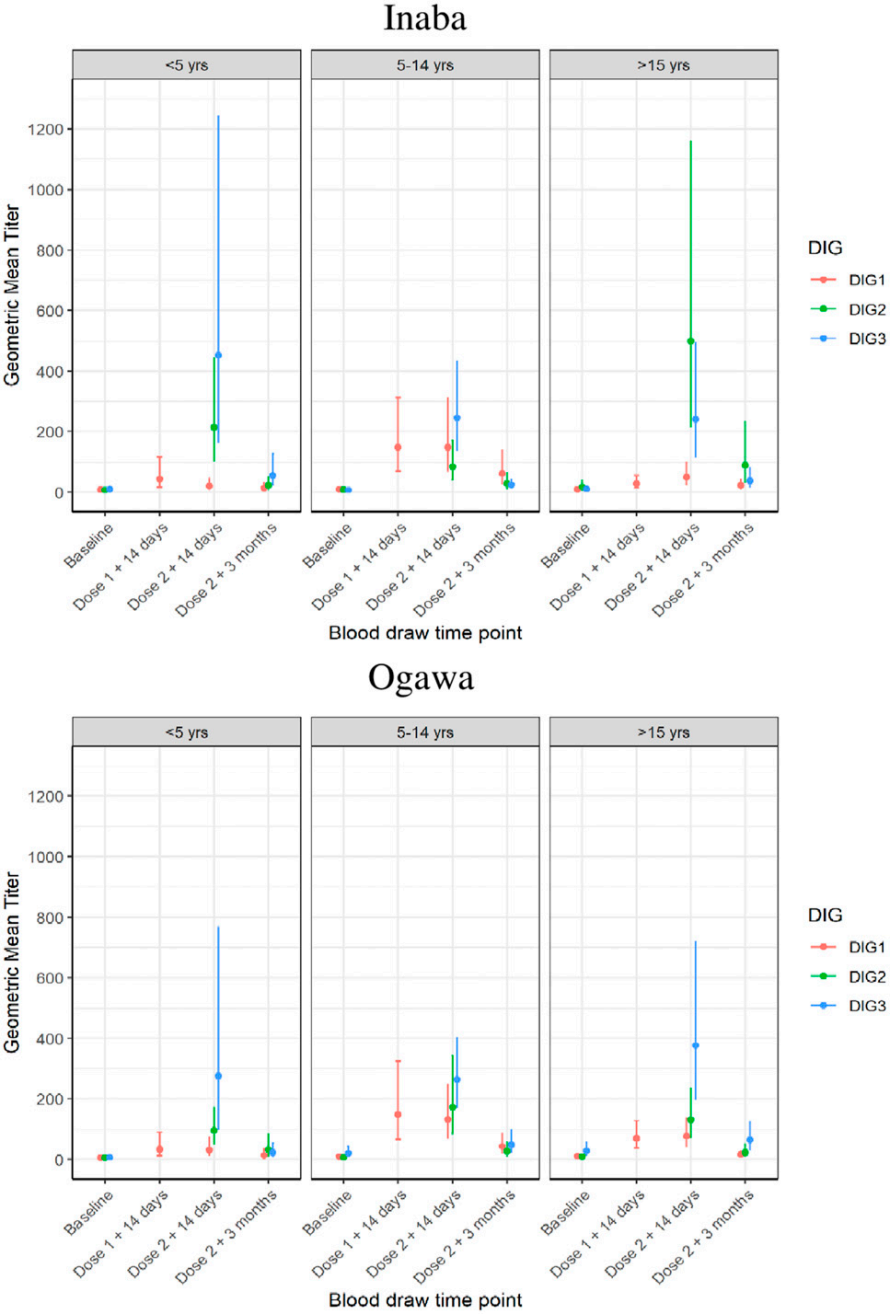
Age stratified Inaba and Ogawa Geometric Mean Titers at baseline and following the first and second dose of oral cholera vaccine. DIG = dose-interval group.

The age-specific vibriocidal GMTs are shown in Figure 4. The same trend for higher GMTs for DIG2 and DIG3 is observed for each of the age strata; however, the sample sizes are too small to make conclusions about differences between the age groups. The vibriocidal response rates for both Inaba and Ogawa were higher for the groups receiving the delayed doses. Table 4 shows the response rates when DIG1 is compared with the groups with a delayed second dose (DIG2 and DIG3 combined).

## DISCUSSION

This study from Cameroon found that extending the dose interval to either 6 or 11.5 months elicited a higher vibriocidal GMT 14 days after the second dose compared with the standard 14-day interval. After a single dose of OCV, the GMT increased among participants in DIT1, but the second dose given 2 weeks later did not increase the GMT further. By contrast, when the second dose was delayed for 6 or 11.5 months, the increase in vibriocidal GMT was significantly higher than when the second dose was given after 2 weeks. Furthermore, by delaying the second dose, there were two periods of the year, one after each dose, with an elevated vibriocidal titer; however, each of these periods with an elevated titer were relatively brief. Three months after vaccination, for each of the groups, the GMTs fell to lower levels, but the titers were still significantly higher that the baseline GMT. Thus, the study showed that the vibriocidal GMTs 2 weeks after the second dose were not inferior to the standard interval and in fact were superior to the standard dose interval. In addition to the higher GMTs with the delayed second dose, the vibriocidal response rate was also higher if the second dose was delayed.

The recommendation for a second dose after 2 weeks was based on results of the pivotal study in Kolkata that used this 2-week dose interval and found this strategy to be protective for 5 years.^23^ When that study was designed, it was assumed that protection required at least two doses.^24^ This assumption was based on an earlier study from Bangladesh with an earlier version of the vaccine in which a single dose was found not to be efficacious. Furthermore, it was assumed that an effective vaccination strategy needed to be rapid to control outbreaks quickly. A strategy with a 2-week interval was as rapid as was possible considering immunological and logistical considerations. Subsequently, a single dose with the current version of vaccine was found to be effective for at least a year,^25^ and for many situations, it was estimated that an initial single dose, to be followed later by a second dose, is an appropriate strategy.^14^ Thus, it seems that a delay in administering the second dose will not compromise field effectiveness of OCV. This is especially true considering the limited supply of vaccine when a single dose, given to more people, is projected to prevent more cases than two doses to fewer people.^14^

A dose-interval study with similar design conducted in rural Zambia showed that extending the interval between doses to 6 months stimulated GMT vibriocidal titers that were not inferior to that stimulated by the 2-week schedule.^17^ The study also found that some subgroups demonstrated a higher rise in Ogawa GMTs, but overall, the rise in GMTs were similar. Thus, both the Zambian and the Cameroon studies show noninferiority for an extended dose interval. Another study done in Kolkata, India, found no statistically significant differences between a 28-day dose-interval group and a 14-day dose-interval group.^15^ We do not have an explanation for the clearly higher GMTs in this study in Cameroon after a delayed second dose, but it is reassuring that neither the Zambian study nor this one in Cameroon found a delayed second dose to be inferior. One potential explanation may be the severe cholera epidemic that occurred in 2010–2012, which may have primed the population in Douala, but this would potentially have affected only the older age groups.

There is a potential immunological explanation for the higher vibriocidal response after a delayed second dose. The first dose of vaccine stimulates an intestinal immune response, which is maximal at about 9 to 14 days after vaccination.^26^ When the second OCV dose is given at this time, the intestinal antibodies stimulated by the first dose might bind and neutralize the vaccine antigens and block the response to the second dose. Studies from South Sudan found no additional seroconversion after a second dose when the second dose was given 3 weeks after the first dose,^27^ and studies from Kolkata found that the second dose given 2 weeks after the first dose did not increase the vibriocidal responses.^15^ By waiting for the initial intestinal immune response to lessen, one might avoid an immune interference from the first dose.

The study did not have sufficient power for age-stratified analysis due to a small sample size, but higher vibriocidal responses were observed in the DIG2 and DIG3 groups among participants aged 1 to 4 years. Although not statistically significant, the 1 to 4 years age group had a lower GMT and lower seroconversion rate at 14 days post–dose 1 or dose 2 compared with the other two groups. The higher response rates with the delayed second dose for youngest children is of potential importance because vaccine efficacy and protection is lower in the age group < 5 years. The potential benefit of delaying the second dose for young children needs to be balanced against the longer interval between doses, which leaves young children more vulnerable until they receive the second dose. For preventive campaigns designed to prevent future outbreaks (but not an ongoing outbreak), this issue is less important. However, during an outbreak, when active cholera transmission is ongoing, a shorter interval between doses may be favored.

In a single-dose efficacy study and its follow-up study in Dhaka, Bangladesh, one dose of OCV was not efficacious for children ≤ 5 years.^28^ On the basis of a systematic review, young children < 5 had a lower seroconversion rates after a single dose, but this did increase after a second dose given 2 weeks later.^29^ In another Bangladesh study, young children who had received a single dose of OCV developed an enhanced vibriocidal response when given OCV 3 years later compared with others who had received a placebo 3 years earlier, suggesting that the earlier dose had primed for a later booster response.^16^ The increased responses seen in this Cameroon study suggests that the delayed response may also stimulate an augmented response in this young age group. However, this observation will need to be confirmed with larger sample numbers of young children.

These findings should be considered when considering logistical and practical implications of delaying the second dose. When vaccine campaigns are carried out, the campaign attempts to provide vaccine to all persons ≥ 1 year residing in the designated area. Thus, the two vaccine rounds may reach somewhat different individuals in the two rounds if there is movement of people in and out of the area. With a longer delay, there is a higher chance that some persons will have moved away and others will have moved into the area. This increases the number of people who will receive at least one dose, but also increases the number who will receive only a single dose. For extremely mobile populations (e.g., refugees), the control program will need to consider the movement of people and may have to consider a third round.

OCV responded more vigorously if they had received OCV 3 years earlier.^16^ A booster response with a similar killed oral vaccine for enterotoxigenic *Escherichia coli* was seen in subjects who had received the vaccine 1 to 2 years earlier.^30^ In that study, the previously vaccinated subjects had an earlier and an enhanced antibody response detected using the ALS assay (antibody in lymphocyte secretions) compared with naive vaccinees. The oral cholera vaccine is assumed to protect primarily through activation of local intestinal immunity, and the vaccine protective antigen is thought to be LPS; thus, a booster response to this oral vaccine is different from booster responses to injectable antigens. Nevertheless, it is worth noting that other vaccines including human papilloma virus,^31^ *Haemophilus influenzae* Type B, and hepatitis B^32^ appear to benefit when the final dose is given after a long interval of 6 to 12 months, or even longer. A longer dose interval also appears to enhance the immune response to COVID vaccines.^33^

The study had several strengths that should be highlighted. First, the participants were studied over a long period with multiple follow-up serum specimens allowing for evaluation of the persistence of vibriocidal titers. Second, the study was conducted in an area at high risk for cholera, but unlike areas in Asia where similar studies have been carried out, no cases were reported in recent years, and none were seen during the conduct of the study when such cholera infections might have confounded the results. Third, because age is an important variable, this study included a similar number of subjects in the different age strata.

There were also some limitations. The sample size was determined based on a comparison of the primary outcomes between the three DIGs but was not sufficient to conduct this comparison for the age-specific strata. Second, we did not collect serum from participant in DIG2 and DIG3 2 weeks after the first dose because we wanted to limit the number of blood collections and assumed the randomization would provide data to define the response 2 weeks after the first dose. Also, the slightly lower proportion of male subjects in DIG3 was noted. Some studies have found a sex difference in immune responses to other injected vaccines,^34^^,^^35^ but it seems unlikely that this difference by sex would account for the differences seen in this study. The children aged 1 to 4 years were generally well nourished, which may limit these findings if extended to other populations where malnutrition is more prevalent. Finally, this study was not intended to monitor cholera cases, and one cannot extend our findings to determine a dose interval that will be most efficacious for reducing the rates of cholera; however, the data should provide reassurance that a delayed dose interval between the first and second doses can be considered an acceptable strategy.

There are several implications from this study. For example, in regions with a predictable cholera seasonality (e.g., Burundi^36^), a single-dose OCV campaign could be carried out shortly before the expected season and then provide the second dose a year later, again before the cholera season. Another implication relates to plans for revaccinating cholera hotspots. Currently, the recommendation suggests revaccination 3 to 5 years after the first campaign to maintain protection. This recommendation assumes two doses will be needed for revaccination; however, if these repeat campaigns target populations already primed by the earlier campaigns, a single dose may be sufficient for the revaccination. One indicator of a booster response is a rapid and vigorous response with revaccination after 3 years,^16^^,^^37^ and this is now being evaluated in Cameroon.

## CONCLUSION

This study found that extending the dose interval to 6 or 11.5 months elicits higher vibriocidal antibody titers 14 days after the second dose compared with the standard 14-day interval, and this should allow increased flexibility regarding timing of the second dose for programs implementing vaccine campaigns. Future studies are needed, using case–control or test-negative designs, to monitor vaccine effectiveness of delayed dose intervals in countries where the second doses were delayed. Second, future studies are needed with larger groups of young children to evaluate whether a longer dose interval will improve immune responses in this vulnerable age group. Finally, if these findings are further validated, a recommendation for a change in dose schedule may be considered for OCV.
